# Incidence and risk factors of fasting hyperglycaemia following first-attack acute pancreatitis before discharge: a retrospective study

**DOI:** 10.1186/s12876-023-02775-7

**Published:** 2023-06-12

**Authors:** Chengsi Liu, Qiao Shi, Xiaoyi Zhang, Enfu Xue, Hanjun Li, Weixing Wang

**Affiliations:** 1 Department of General Surgery, Renmin Hospital of Wuhan University, Wuhan, 430060 Hubei Province China; 2 Department of Pancreatic Surgery, Renmin Hospital of Wuhan University, Wuhan, 430060 Hubei Province China; 3 Department of Critical Care Medicine, Zhongnan Hospital of Wuhan University, Wuhan, 430060 Hubei Province China

**Keywords:** Acute pancreatitis, Fasting hyperglycaemia, Impaired fasting glucose, Diabetes, Risk factors, Triglycerides

## Abstract

**Background:**

Pancreatic endocrine insufficiency is more likely to occur after acute pancreatitis (AP), but the risk factors affecting pancreatic endocrine function remain controversial. Therefore, exploring the incidence and risk factors of fasting hyperglycaemia following first-attack AP is important.

**Methods:**

Data were collected from 311 individuals with first-attack AP without previous diabetes mellitus (DM) or impaired fasting glucose (IFG) history treated in the Renmin Hospital of Wuhan University. Relevant statistical tests were performed. A two-sided p-value < 0.05 was considered statistically significant.

**Results:**

The incidence of fasting hyperglycaemia in individuals with first-attack AP was 45.3%. Univariate analysis showed that age (χ^2^ = 6.27, *P* = 0.012), aetiology (χ^2^ = 11.184, *P* = 0.004), serum total cholesterol (TC) (χ^2^ = 14.622, *P* < 0.001), and serum triglyceride (TG) (χ^2^ = 15.006, *P* < 0.001) were significantly different between the hyperglycaemia and non-hyperglycaemia groups (*P* < 0.05). The serum calcium concentration (Z=-2.480, *P* = 0.013) was significantly different between the two groups (*P* < 0.05). Multiple logistic regression analysis showed that age- ≥60 years (*P* < 0.001, OR = 2.631, 95%Cl = 1.529–4.527) and TG ≥ 5.65 mmol/L (*P* < 0.001, OR = 3.964, 95%Cl = 1.990–7.895) were independent risk factors for fasting hyperglycaemia in individuals with first-attack AP (*P* < 0.05).

**Conclusions:**

Old age, serum triglycerides, serum total cholesterol, hypocalcaemia, and aetiology are associated with fasting hyperglycaemia following first-attack AP. Age ≥ 60 years and TG ≥ 5.65 mmol/L are independent risk factors for fasting hyperglycaemia following first-attack AP.

**Supplementary Information:**

The online version contains supplementary material available at 10.1186/s12876-023-02775-7.

## Background

Acute pancreatitis (AP) is among the most common clinical digestive system diseases. It is an inflammatory disease caused by the abnormal activation of pancreatic enzymes, leading to the digestion of pancreatic tissues. AP has a rapid onset and progression and can develop into moderately severe acute pancreatitis (MSAP) or severe acute pancreatitis (SAP), which has many complications, dangerous conditions, and high mortality [1, 2].

The pancreas, which is an important organ of internal and external secretion, plays an important role in digestion and blood glucose regulation. The main cell components of the endocrine pancreas include β-cells that secrete insulin. It has been reported that 37% of patients develop impaired glucose metabolism two years following the first acute alcoholic pancreatitis [3].

Diabetes mellitus (DM) is a universal chronic endocrine disease characterised by elevated blood glucose levels [4]and is an important cause of coronary heart disease, renal failure, lower limb ischaemia, blindness, and amputation [5–7]. AP-related DM is gaining recognition as a sequela of AP. While it is more common in patients with necrotizing or severe AP, patients with mild AP are also at increased risk for developing this complication [8]. Shen et al. retrospectively analysed 2966 patients with AP and found that new-onset DM in patients after AP was 2.5 times higher than that in healthy individuals [9]. The study by Lee et al. on 3187 individuals with AP showed that the incidence of new-onset DM in patients after AP was 2.1 times higher with 709,259 random general population as a control [10]. Some studies have found that risk factors for developing DM in individuals with primary AP include hyperlipidaemia, increased bedside index of severity in acute pancreatitis score, and non-biliary pancreatitis [11]. SAP, alcoholic AP and acute necrotizing pancreatitis (ANP) were associated with increased incidence of DM after AP [12].These studies suggest that pancreatic endocrine deficiency is more likely to occur after AP; however, the risk factors affecting pancreatic endocrine function remain controversial. Admission and persistent stress hyperglycaemia during the first week of admission worsens clinical outcomes of AP patients [13], there was a significant relationship between stress hyperglycaemia and adverse clinical outcomes [14]. Therefore, this study retrospectively analysed the medical records of 311 individuals with the first episode of AP in the Department of Pancreatic Surgery of our hospital to explore the possible risk factors of fasting hyperglycaemia and to provide evidence for the prevention and treatment of impaired fasting glucose (IFG) or DM after a first-attack AP.

## Methods

Clinical data of 311 individuals with AP hospitalised in the Department of Pancreatic Surgery, Renmin Hospital of Wuhan University, from January 2018 to November 2019, were retrospectively collected. All procedures were conducted in accordance with the relevant guidelines and regulations. Patients who met the inclusion and exclusion criteria were included in the analysis. The time from onset to admission was no more than 7 days.

This study was conducted in accordance with the principles of the Helsinki Declaration. Due to the retrospective characteristics of the study from January 2018 to November 2019, informed consent was waived and the study was approved by the ethics committee of the Renmin Hospital of Wuhan University (Registration Number: WDRY 2020-K177).

### Definitions

Those who met any two of the following three criteria were considered to have been diagnosed with AP: (1) abdominal pain consistent with AP; (2) serum amylase or lipase activity three times higher than the normal upper limit; and abdominal imaging examination consistent with AP imaging changes. (3) IFG and DM diagnosed according to the World Health Organization (WHO) criteria [15] (IFG: 6.1 mmol/L ≤ fasting plasma glucose (FPG) < 7.0 mmol/L; DM:FPG ≥ 7.0 mmol/L or random glucose ≥ 11.1 mmol/L). Based on diagnostic criteria [15], FPG ≥ 6.1 mmol/L was defined as fasting hyperglycaemia in this study.

### Exclusion criteria

① Chronic pancreatitis or acute attack of chronic pancreatitis; ② recurrent acute pancreatitis; ③ DM or IFG diagnosed before AP. ④ Chronic gastrointestinal history before AP; ⑤ family history of DM; ⑥ history of pancreatic tumour; and ⑦ age < 18 years.

### Data collection

The clinical data collected included the following: sex, age, cause of AP, grades of severity, hospital inspection results in 48 h (serum total cholesterol (TC), serum triglyceride (TG), serum albumin (ALB), white blood cell (WBC) count, alanine aminotransferase (ALT), aspartate aminotransferase (AST), serum levels of total bilirubin (TBIL), direct bilirubin (DBIL), urea, creatinine, and serum Ca^2+^ concentration).

### Grouping of data

(1) According to the Atlanta AP diagnostic criteria [16], individuals were divided into two groups: MAP and MSAP + SAP. (2) Individuals were divided into two groups according to sex. (3) Age (60 years). (4) According to the cause of AP, individuals were divided into three groups: biliary, hyperlipidaemia, and other. The diagnostic principle of biliary AP is determined after excluding alcohol, drug - induced, hyperlipidaemia and other causes of acute pancreatitis, according to the gallbladder or biliary sludge detected by EUS, AUS, CT or MRCP, or imaging showed no gallbladder or biliary sludge, but accompanied by elevated liver function indicators such as TBIL, AST and ALT. The diagnostic criteria for hyperlipidaemia AP are as follows: serum TG > 11.3 mmol/L and other causes such as biliary stones, alcohol and drugs are excluded, or 5.6 mmol/L < serum TG ≤ 11.3 mmol/L with a history of hyperlipidaemia were diagnosed. Individuals who did not conform to the first two diagnoses were included in the other group. (5) According to the Chinese guidelines on prevention and treatment of dyslipidaemia in adults in 2007 [17], TC = 6.21 mmol/l and TG = 5.65 mmol/l were used as the boundary. (6) According to the diagnostic criteria for systemic inflammatory response syndrome [18], a WBC count of 12 × 10^9^ /L was used as the boundary. (7) According to WHO diagnostic criteria for DM [15], individuals were divided into two groups with a blood glucose level of 6.1 mmol/L as the boundary.

### FPG measurement

FPG was detected using glucose oxidase. 3.0 ml of elbow venous blood was collected from all individuals with AP 12 h after fasting. A Siemens ADVIA® XPT automatic biochemical analyser and supporting reagents were used for detection. The average FPG values of the last two measurements (the day of and the day before discharge) were statistically analysed.

### Statistical analysis

SPSS 22.0 statistical software was used for analysis. The counting data were represented by the number of cases and percentage (n, %) and were compared using a single factor analysis (χ^2^ test). The distribution characteristics of the measurement data were tested using the Shapiro-Wilk method. The Mann–Whitney U test was used for skewness distribution data, which were represented by the median and interquartile range (IQR). Multiple imputations were performed by using 20 iterations to assure convergence for missing values (Supplementary Table 1). Statistically significant variables were included in the multivariate binary logistic regression analysis, and a stepwise regression method was used. All statistical tests were two-sided, and a *p-*value < 0.05 was considered statistically significant.

## Results

### Fasting hyperglycaemia (FPG ≥ 6.1 mmol/L) occurred in 45.3% of the first episode of AP individuals on the day of discharge

**Fig. 1:**
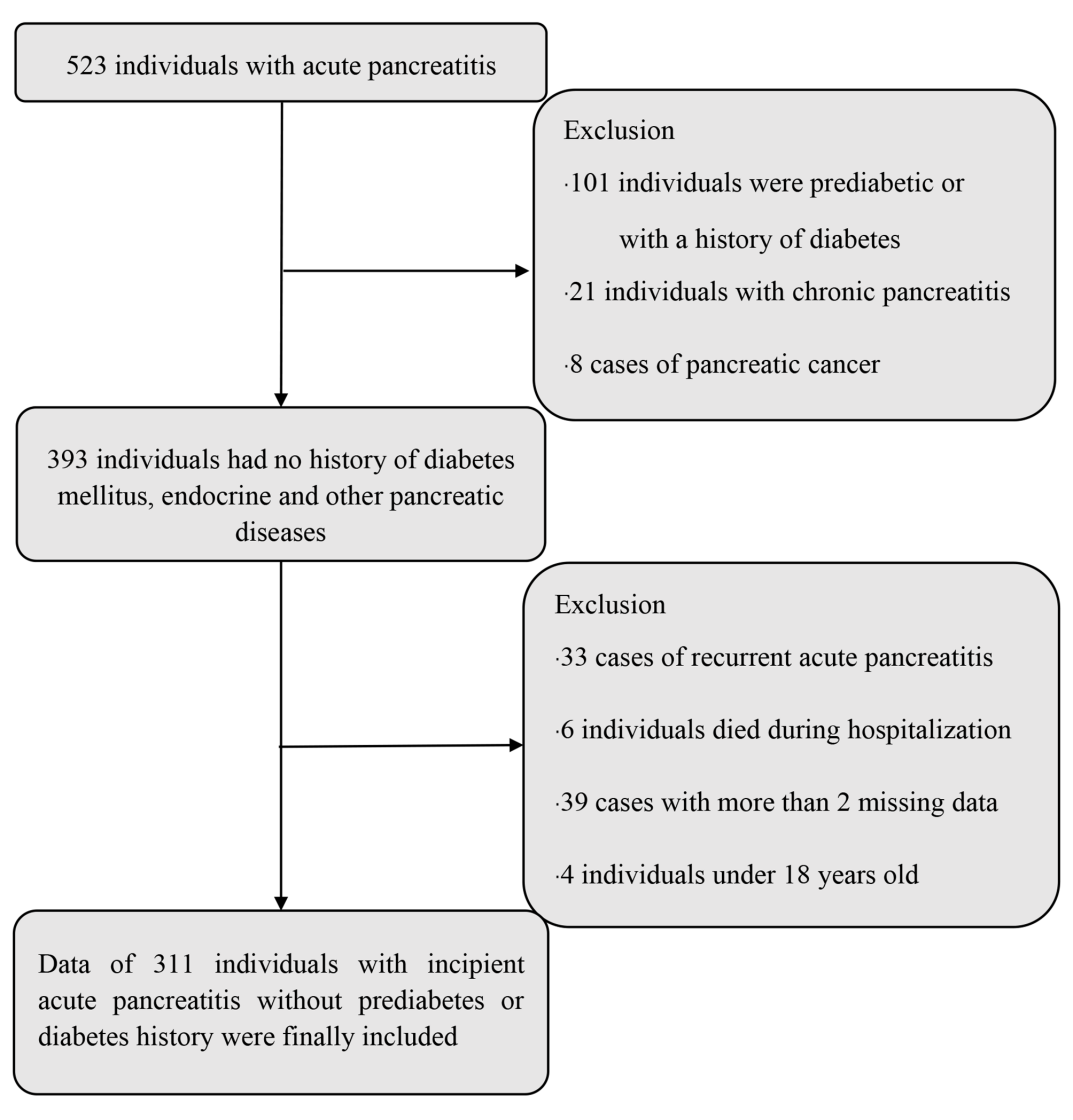
Study flowchart

Among the 311 individuals with the first episode of AP (Figs. 1), 141 had FPG ≥ 6.1 mmol/L on the day of discharge, and the incidence of fasting hyperglycaemia was 45.3%. The median IQR blood glucose levels was 5.88 mmol/L (IQR: 5.0–7.3 mmol/L). In addition, six patients suffered SAP, all of whom died during hospitalization and were excluded from the study.

**Fig. 2:**
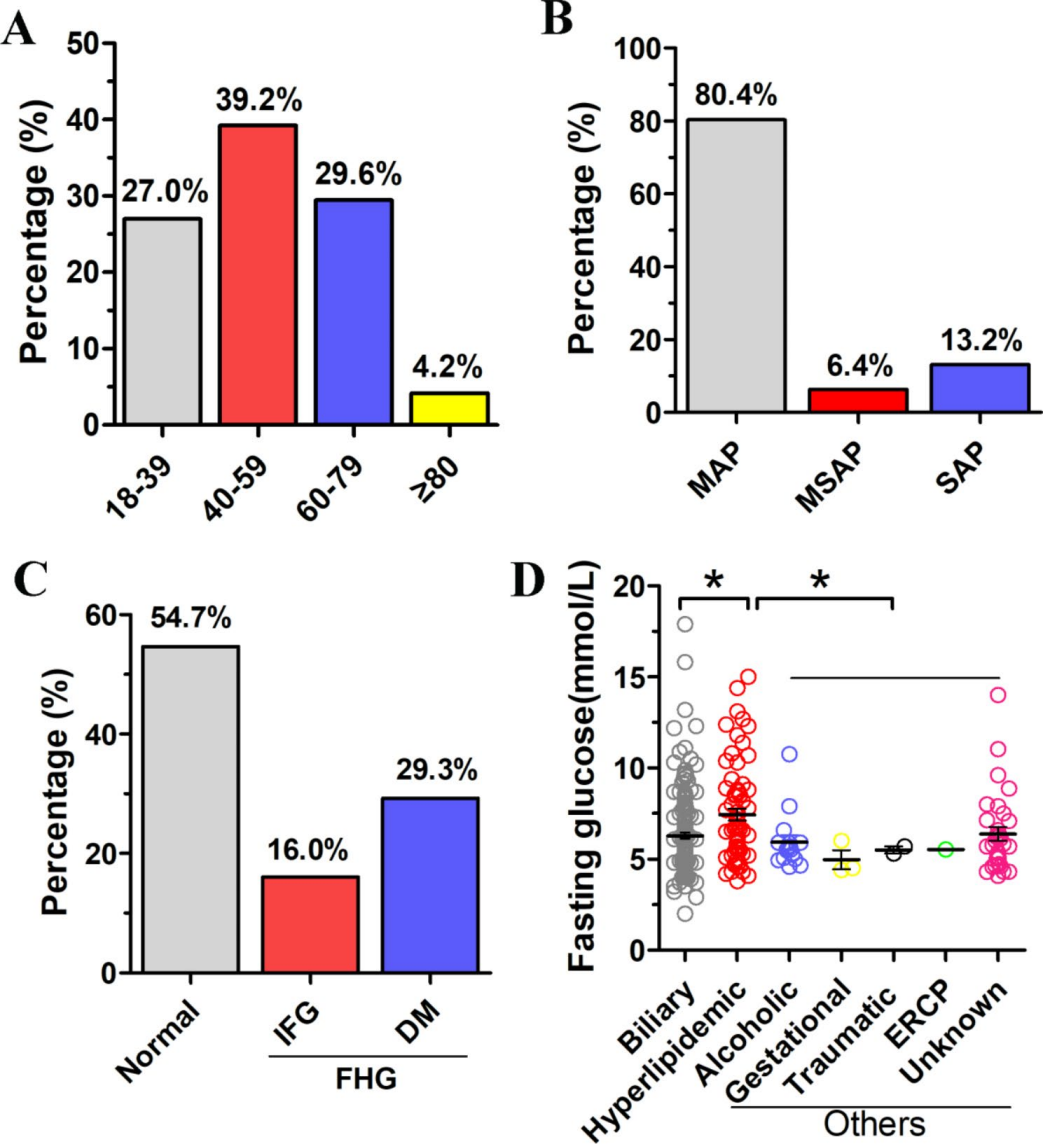
Age, severity, blood glucose and etiology distribution of individuals with acute pancreatitis. (A) The proportions of people of different ages. (B) The proportions of the MAP, MSAP and SAP. (C) The proportion of hyperglycaemia, IFG and DM. (D) The boxplot of blood glucose distribution in individuals with AP under different etiologies. (MAP:mild acute pancreatitis, MSAP:moderately severe acute pancreatitis, SAP:severe acute pancreatitis, IFG:impaired fasting glucose, DM:diabetes mellitus, FHG:fasting hyperglycemia, ERCP:endoscopic retrograde cholangiopancreatography.) *P < 0.05 vs. Hyperlipidemic group

The results revealed more men than women, with a range of ages from 18 to 88 years, a median (IQR) age of 52 years (IQR: 38–64 years). MAP, MSAP, and SAP accounted for 80.4, 6.4, and 13.2%, respectively. The proportion of biliary AP was significantly higher than that of hyperlipidaemic and other etiological groups. The cause of AP includes alcohol consumption, pregnancy, trauma, and endoscopic retrograde cholangiopancreatography. After the initial onset of AP, the proportion of individuals in the hyperglycaemia group was 45.3%, of which IFG accounted for 16% and DM 29.3% (Fig. 2; Table 1).

**Table 1:** Clinical characteristics of 311 individuals with first-attack acute pancreatitis

Characteristics	Avg	Median(IQR)
**FPG on discharge(mmol/L)**	6.52	5.88(5,7.29)
**Age**	51.4	52(38,64)
**TC(mmol/L)**	5.04	4.58(3.76,5.54)
**TG(mmol/L)**	3.6	1.46(0.86,3.78)
**Alb(g/L)**	37.7	37.6(33.6,42.3)
**WBC count(×10^9/L)**	11.69	11.23(8.35,14.32)
**ALT(U/L)**	125.6	35(18,150)
**AST(U/L)**	122.6	35(23,113)
**TBIL(umol/L)**	28.2	19.4(12.5,30.4)
**DBIL(umol/L)**	14.5	6.2(3.7,13.6)
**Urea(mmol/L)**	5.7	5(4.1,6.4)
**Cre(mmol/L)**	72.5	63(50,76)
**Serum Ca** ^**2+**^ **(mmol/L)**	2.15	2.18(2.06,2.29)

(FPG:Fasting plasma glucose, TC: Serum total cholesterol, TG: Triglyceride, Alb: Albumin, WBC:White blood cell, ALT :Alanine aminotransferase, AST:Aspartate aminotransferase, TBIL:Total bilirubin, DBIL:Direct bilirubin.)

### Age, cause of AP, TC, serum TG and serum Ca^2+^concentrations were correlated with fasting hyperglycaemia in individuals with the first episode of AP

The results showed that fasting hyperglycaemia occurred more frequently in people aged ≥ 60 years (χ^2^ = 6.27, P = 0.012). The incidence of hyperglycaemia in the hyperlipidaemia group was significantly higher than that in the biliary and other etiological groups (χ^2^ = 11.184, P = 0.004). The incidence of fasting hyperglycaemia was higher when TC was ≥ 6.21 mmol/L or serum TG was ≥ 5.65 mmol/L (χ^2^ = 14.622, *P* < 0.001; χ^2^ = 15.006, *P* < 0.001). That is, age, aetiology, TC, and TG levels were correlated with fasting hyperglycaemia in people after a first-attack AP (Table 2).

**Table 2:** Univariate analysis results of clinical characteristics

Characteri-stics	Group	FPG ≥ 6.1 mmol/L, N (%)	FPG < 6.1 mmol/L, N (%)	*χ* ^*2*^	*P*
**Sex**	Male	74 (52.5)	100 (58.8)	1.258	0.262
	Femal	67 (47.5)	70 (41.2)		
**Age**	≥ 60	58 (41.1)	47 (27.6)	6.27	0.012*
	< 60	83 (58.9)	123 (72.4)		
**Grades of** **severity**	MAP	107^a^ (76.1)	143^a^ (84.2)	3.318	0.190
	MSAP	11^a^ (7.7)	9^a^ (5.3)		
	SAP	23^a^ (16.2)	18^a^ (10.5)		
**Etiologies** **of AP**	Biliary	80^a^ (56.7)	110^a^ (64.7)	11.184	0.004*
	Hyperlipidaemic	42^b^(29.8)	25^b^ (14.7)		
	Other	19^a^ (13.5)	35^a^ (20.6)		
**TC**	≥ 6.21 mmol/L	35(24.8)	15 (8.8)	14.622	< 0.001*
	< 6.21 mmol/L	106(75.2)	155 (91.2)		
**TG**	≥ 5.65 mmol/L	39 (27.7)	18(10.6)	15.006	< 0.001*
	< 5.65 mmol/L	102 (72.3)	152 (89.4)		
**ALB**	≤ 30 g/L	20 (14.2)	13 (7.6)	3.473	0.062
	> 30 g/L	121 (85.8)	157 (92.4)		
**WBC count**	≥ 12 × 10^9/L	67 (47.5)	66(38.8)	2.380	0.123
	< 12 × 10^9/L	74 (52.5)	104 (61.2)		

Table 2 : Table 2 shows the univariate analysis results of different clinical features and patients with fasting hyperglycaemia. In the comparison between groups larger than 3, “a” and “b” are regarded as significant differences between the two groups, while “a” and “a” are regarded as insignificant differences between the two groups.*P < 0.05.

(FPG:Fasting plasma glucose, MAP:mild acute pancreatitis, MSAP:moderately severe acute pancreatitis, SAP:severe acute pancreatitis, TC: Serum total cholesterol, TG: Triglyceride, ALB: Albumin, WBC:White blood cell.)

Some indicators (ALT, AST, TBIL, DBIL, urea, creatinine, and serum Ca^2+^) were analysed using the Shapiro-Wilk test, and the above data were skewed distribution measurements. The results showed no significant differences between the discharged fasting hyperglycaemia and control groups (*P* > 0.05). Only the serum Ca^2+^ concentration showed a statistically significant difference between the two groups (*P* < 0.05) (Table 3). That is, serum Ca^2+^ concentration is associated with fasting hyperglycaemia after the initial onset of AP.

**Table 3:** U test results of clinical characteristics

Characteristics	Fasting hyperglycaemia N = 141(FPG ≥ 6.1mmol/L)	Control N = 170(FPG < 6.1mmol/L)	Z	*P*
**ALT(U/L)**	30(18,106)	39.5(18,194.3)	-1.490	0.136
**AST(U/L)**	33(21,102)	36.5(24,124.3)	-1.225	0.221
**TBIL(umol/L)**	19.3(12,27.7)	19.6(12.9,35.5)	-0.970	0.332
**DBIL(umol/L)**	6(3.25,11)	6.3(3.8,15.88)	-1.169	0.243
**Urea(mmol/L)**	5.3(4.1,6.7)	4.8(4.1,6.3)	-1.008	0.313
**Cre(mmol/L)**	63(48,82)	63(52,74)	-0.369	0.712
**Serum Ca** ^**2+**^ **(mmol/L)**	2.15(2.03,2.28)	2.22(2.08,2.30)	-2.480	0.013^*^

Table 3 : The U test results of clinical characteristics showed that serum calcium ion levels were associated with fasting hyperglycaemia.*P < 0.05. (FPG:Fasting plasma glucose, ALT :Alanine aminotransferase, AST:Aspartate aminotransferase, TBIL:Total bilirubin, DBIL:Direct bilirubin.)

### Age ≥ 60 years and TG ≥ 5.65 mmol/L were independent risk factors for fasting hyperglycaemia in first episode of AP individuals

The multi-factor binary logistic regression results showed that age ≥ 60 years (OR = 2.631, 95%CI = 1.529–4.527) was an independent risk factor for fasting hyperglycaemia (FPG ≥ 6.1 mmol/ L) (*P* < 0.05), compared with individuals with aged < 60 years, the possibility of fasting hyperglycaemia after first-attack AP was 2.631 times. TG ≥ 5.65 mmol/L (OR = 3.964, 95%CI = 1.990–7.895) was an independent risk factor for fasting hyperglycaemia (FPG ≥ 6.1 mmol/ L) in first episode of AP individuals (*P* < 0.05), compared with individuals with TG < 5.65 mmol/L, the possibility of fasting hyperglycaemia was 3.964 times (Table 4).

**Table 4:** Multiple logistic regression analysis results

Characteristics	β	S.E.	Wald	df	OR	95% confidence interval		*P*
						Lower Bound	Upper Bound	
**Age(≥60)**	0.967	0.277	12.204	1	2.631	1.529	4.527	<0.001^*^
**Gender**	-0.181	0.275	0.436	1	0.834	0.487	1.429	0.509
**Etiologies of AP**								
**Biliary**			2.949	2				0.229
**Hyperlipidaemic**	-0.420	0.396	1.123	1	0.657	0.303	1.428	0.289
**Other**	0.389	0.380	1.045	1	1.475	0.700	3.109	0.307
**Grades of severity**								
**MAP**			0.285	2				0.867
**MSAP**	-0.271	0.533	0.258	1	0.763	0.268	2.169	0.612
**SAP**	0.033	0.441	0.006	1	1.033	0.435	2.452	0.941
**TC(**≥ **6.21mmol/L)**	0.483	0.498	0.938	1	1.620	0.610	4.302	0.333
**TG(**≥ **5.65mmol/L)**	1.377	0.352	15.347	1	3.964	1.990	7.895	<0.001^*^
**Serum Ca** ^**2+**^	0.553	0.613	0.814	1	1.739	0.523	5.782	0.367

Table 4: Meaningful results of univariate analysis were included in multivariate regression analysis, and the results showed that age ≥ 60 and TG ≥ 5.65mmol/l were independent risk factors for fasting hyperglycaemia, while others were not.*P < 0.05. (MAP:mild acute pancreatitis, MSAP:moderately severe acute pancreatitis, SAP:severe acute pancreatitis, TC: Serum total cholesterol, TG: Triglyceride.)

## Discussion

AP is characterised by acute inflammation of the pancreas and destruction of tissue acinar cells. The mortality for pancreatitis is approximately 1% overall; approximately 80% of patients with acute pancreatitis have mild to moderately severe disease (absence of organ failure > 48 h). However, one-fifth of patients’ conditions will progress to severe disease, with a mortality rate of approximately 20% [19]. Hyperglycaemia has been reported to aggravate acute pancreatitis [20]. Hyperglycaemia after AP refers to a temporary increase in blood glucose concentration in people without a history of IFG or DM after acute physiological injury [21]. Presently, the phenomenon of elevated blood glucose levels after AP is rarely considered. When patients in the acute stage of the disease are admitted to the hospital, the stress state may lead to hyperglycaemia, and the FPG level is greatly affected. However, their condition stabilises and the FPG is less affected when they recover from the acute-stage inflammation. The fasting blood glucose level before the patient is discharged can reflect the level of islet function of individuals. Therefore, exploring the factors affecting fasting blood glucose at discharge is crucial.

Pancreatic endocrine function injury after AP has gradually attracted attention. Petrov et al. found that islet cell damage and insulin resistance are important causes of hyperglycaemia in people with AP [22]. Patients with AP developed DM after discharge from hospital with a frequency of approximately 23% [12]. Tu et al. found that the severity of AP, area of pancreatic tissue necrosis, and insulin resistance were important risk factors for the occurrence of DM after AP, and the incidence of IFG + DM after AP was approximately 59.25% [23]. Most of these studies involved the long-term follow-up of individuals. After the follow-up, some individuals developed IFG or DM. Therefore, it is particularly important to identify the relevant indicators that indicate the development of IFG and DM in people with early-stage disease. This study retrospectively analysed the clinical data of 311 individuals with the first episode of AP; the incidence of fasting hyperglycaemia on the day of discharge after first-attack AP was 45.3%.

Current studies suggest that IFG or IGT after diabetes and AP is related to age [24]. Some studies have shown that male individuals with AP aged < 45 years have the greatest risk of diabetes after AP [25]. Our results are consistent with the common observation of hyperglycaemia in middle-aged and elderly individuals, possibly because of the lack of functional islet β cells associated with AP inflammation in elderly people. The function of islet β cells gradually declines with age [26]. The influence of age on fasting hyperglycaemia after first-attack AP is similar to the conclusions of some researchers, and the reason for the difference may be associated with the specificity of the regional distribution of individuals with AP. Moreover, there are regional differences in the incidence of diabetes. DM incidence is higher in Asian and Middle Eastern countries than in Western countries [27]. Several studies have suggested that genetic predisposition, socioeconomic status, culture, and lifestyle may be responsible for this difference [28, 29].

Sex is not a reported factor influencing IFG or DM after AP [30, 31], which is consistent with our results. However, our study identified more men than women with AP, which may be related to men’s behavioural habits, such as smoking and alcoholism [32]. Our results revealed that the biliary origin was the highest in both men and women with AP. Biliary tract disease remains the main cause of AP in China, and AP caused by hyperlipidaemia is more common in men than it is in women, which may be related to the dietary structure and lifestyle of both men and women [33].

Biliary tract disease, hyperlipidaemia, and excessive alcohol consumption are three important factors associated with the occurrence of AP [2]. Additionally, smoking may increase the risk of AP in the population and is an independent risk factor [34]. In several large retrospective studies, type II diabetes increased the risk of AP by 1.86 to 2.98-fold [35, 36]. More cases of fasting hyperglycaemia have been reported after AP in people with hyperlipidaemia. Hyperlipidaemia blocks pancreatic microvascular circulation and further affects pancreatic secretory function, which is prone to elevated blood glucose levels. Therefore, attention should be paid to lipid indicators, such as cholesterol and triglycerides, in individuals with AP. Although the aetiology was not an independent risk factor for fasting hyperglycaemia after the initial onset of AP, subsequent studies are needed to refine the classification and analysis of lipid indicators in people with hyperlipidaemia.

The severity of AP depends on the extent of the local injury in and around the pancreas, as well as systemic injury to remote organs. The proportion of patients with different severity of AP varies across different studies. Population-based studies have shown the proportion of SAP between 8% and 20% [37]. Moreover, the proportion of patients with organ failure in large series of patients from tertiary care hospitals reached up to 40% [37]. In a prospective study of 1655 people based on the Revision of Atlanta classification, MAP, MSAP, and SAP accounted for 65%, 28.16%, and 6.83% [38]. Another study on 1186 patients with acute pancreatitis reported organ failure in 240 of 639 patients (38%) with necrotizing pancreatitis [39]. The possible reason for the small proportion of MSAP and SAP in our study may be that organ failure is a dynamic process that is not limited to the first week and may develop at any stage of the disease. Conversely, ours was a single centre study, like previous studies, and included fewer than 100 patients with organ failure. Moreover, it was performed in tertiary referral centres to which > 40% of patients had been transferred from other hospitals during different phases of the disease, which might have limited the data regarding early-phase organ failure [39].

Some studies have reported that AP severity is not associated with emerging diabetes [25, 30], whereas others [12, 40] have associated SAP with an increased incidence of DM after AP. As reported, no association was found between the duration of organ failure and mortality [39]. Thus, it remains unclear whether persistent organ failure and multiple organ failure are major determinants of severity in AP and any kind of local complication corresponds to worse outcomes.

During AP, the abnormal accumulation of intracellular Ca^2+^ promotes excessive activation of trypsinogen, leading to digestive injury of the pancreas [41]. Ca^2+^ influx occurs in the AP, and increased intracellular Ca^2+^ concentration leads to decreased blood Ca^2+^ concentration and intracellular ATP depletion [42]. After the aggravation of inflammation, the necrotic area of the pancreas was large, necrosis of pancreatic β cells was large, the concentration of calcium ions was significantly reduced, the endocrine function of the pancreas was reduced, and hyperglycaemia occurred after AP. A prospective study on people with AP reported that the average total serum calcium content of MAP, MSAP and SAP were 2.06, 1.88, and 1.75 mmol/L, respectively, and that MSAP or SAP should be considered when the total serum calcium content was lower than 2.06 mmol/L [43]. Our results revealed that fasting hyperglycaemia was associated with serum calcium levels because patients with higher serum calcium levels less commonly had fasting hyperglycaemia during first-attack AP.

The effect of renal function indices on fasting hyperglycaemia in individuals with first-attack AP has rarely been reported. Renal insufficiency and dysfunction are common in AP, especially in individuals with SAP. During SAP, a large amount of fluid flows into the third space, resulting in decreased renal blood perfusion. Renal tubules may be damaged due to blood hypercoagulability and infection, resulting in renal insufficiency or dysfunction [44]. This study identified no significant differences in the urea and creatinine levels between the fasting hyperglycaemia and control groups, which were thus not associated with the occurrence of fasting hyperglycaemia in individuals with first episode of AP.

### Limitations of the study

This was a retrospective study; MAP accounted for a large proportion, and the number of individuals with MSAP and SAP was insufficient. Because of the limited number of cases, our descriptions of the AP aetiological and leukocyte classifications are insufficient; therefore, more cases are needed to refine related classifications and conduct in-depth research. It is too simple to classify the age groups based on clinical experience. Age-based differences in the incidence of hyperglycaemia should be carefully distinguished. Our results suggest age and triglyceride have important effects on AP after fasting blood glucose. More samples in multi-centre prospective clinical studies are required for a more detailed testing index and classification of variables for revealing the incipient AP fasting hyperglycaemia in individuals with mechanisms and identifying more influence factors to resolve.

## Conclusions

Fasting hyperglycaemia (FPG ≥ 6.1 mmol/L) occurred in 45.3% of the individuals with first-attack AP after treatment. Age ≥ 60 years and TG ≥ 5.65 mmol/L were independent risk factors for fasting hyperglycaemia in individuals with first episode of AP. In clinical practice, the occurrence of fasting hyperglycaemia after AP should be considered for people with first episode of AP with no history of IFG or DM, age ≥ 60 years, and TG ≥ 5.65 mmol/L. Clinicians should alert individuals with elevated blood glucose levels and strengthen blood glucose monitoring and management during hospitalisation and early intervention. Concurrently, blood glucose monitoring should be continued after discharge and interventions should be administered in advance for people with pre-DM through dietary regulation and oral hypoglycaemic drug treatment to prevent and cure IFG or DM after AP.

## Electronic supplementary material

Below is the link to the electronic supplementary material.

Supplementary Material 1

## Data Availability

The datasets used and analysed during the current study are available from the corresponding author on reasonable request.

## Abbreviations

ALB: Albumin
ALT: Alanine aminotransferase
AP: Acute pancreatitis
AST: Aspartate aminotransferase
DBIL: Direct bilirubin
DM: Diabetes mellitus
FPG: Fasting plasma glucose
IFG: Impaired fasting glucose
IQR: Interquartile range
MSAP: Moderate severe pancreatitis
SAP: Severe acute pancreatitis
TBIL: Total bilirubin
TC: Serum total cholesterol
TG: Triglyceride
WHO: World Health Organization
