# Supplementary material for: Incidence and risk factors of fasting hyperglycaemia following first-attack acute pancreatitis before discharge: a retrospective study
Source: BMC Gastroenterol. 2023 Jun 12;23:203. doi: 10.1186/s12876-023-02775-7 (PMC10259015; doi:10.1186/s12876-023-02775-7)
Supplement: Supplementary file 1 — Supplementary Material 1 [file 12876_2023_2775_MOESM1_ESM.doc]

**Supplementary material**

**Supplementary Table 1. Imputed variables.**

| **Characteri-stics** | **Group** | **Cases(N, %)**  **Before Imputed** | **Missing cases (N,%)** | **Cases(N, %)**  **After Imputed** |
| --- | --- | --- | --- | --- |
| **Sex** | Male | 174 (55.9) | 0 | 174 (55.9) |
|  | Femal | 137 (44.1) |  | 137 (44.1) |
| **Age** | ≥60 | 105 (33.8) | 0 | 105 (33.8) |
|  | < 60 | 206 (66.2) |  | 206 (66.2) |
| **Grades of**  **severity** | MAP | 250 (80.4) | 0 | 250 (80.4) |
|  | MSAP | 20 (6.4) |  | 20 (6.4) |
|  | SAP | 41 (13.2) |  | 41 (13.2) |
| **Etiologies of AP** | Biliary | 190 (61.1) | 0 | 190 (61.1) |
|  | Hyperlipidaemic | 67 (21.5) |  | 67 (21.5) |
|  | Other | 54 (17.4) |  | 54 (17.4) |
| **TC** | ≥6.21 mmol/L | 46 (16.2) | 27(8.7) | 50 (16.1) |
|  | <6.21 mmol/L | 238 (83.8) |  | 261 (83.9) |
| **TG** | ≥5.65 mmol/L | 52 (18.4) | 28(9.0) | 57 (18.3) |
|  | <5.65 mmol/L | 231 (81.6) |  | 254 (81.7) |
| **ALB** | ≤30 g/L | 33 (10.3) | 1(0.3) | 33 (10.6) |
|  | >30 g/L | 277 (89.7) |  | 278 (89.4) |
| **WBC** | ≥12×10^9/L | 133 (42.9) | 1(0.3) | 133 (42.8) |
|  | <12×10^9/L | 177 (57.1) |  | 178 (57.2) |
| **FPG** | ≥6.1 mmol/L | 131 (45.2) | 11(3.5) | 141 (45.3) |
|  | <6.1 mmol/L | 159 (54.8) |  | 170 (54.7) |
| **ALT**  **AST** | / | 311 | 0  0 | 311 |
|  | / | 311 |  | 311 |
| **TBIL** | / | 311 | 0 | 311 |
| **DBIL** | / | 310 | 1(0.3) | 311 |
| **Urea** | / | 310 | 1(0.3) | 311 |
| **Cre** | / | 310 | 1(0.3) | 311 |
| **Serum Ca^2+^** | / | 311 | 0 | 311 |

(MAP:mild acute pancreatitis, MSAP:moderately severe acute pancreatitis, SAP:severe acute pancreatitis, TC: Serum total cholesterol, TG: Triglyceride, ALB: Albumin, WBC:White blood cell, FPG:Fasting plasma glucose, ALT :Alanine aminotransferase, AST:Aspartate aminotransferase, TBIL:Total bilirubin, DBIL:Direct bilirubin.)
